# Ligature of the Carotid Artery

**Published:** 1862-11

**Authors:** H. Wardner

**Affiliations:** Brigade-Surgeon; U. S. General Hospital, Mound City, Ill.


### CHICAGO MEDICAL EXAMINER.

N. S. DAVIS, M.D., Editor.

VOL. III. NOVEMBER, 1862. NO. 11.

### Original Contributions.

### ARTICLE XXXV.

### LIGATURE OF THE CAROTID ARTERY.

By H. WARDNER, M.D., Brigade-Surgeon.

U. S. General Hospital,
Mound City, Ill., *Nov.*, 30*th*, 1862.

Mr. Editor:—I send you the following report of a case of wound of the Carotid Artery, its ligation, and result, thinking it may be of some interest to your readers.

On the evening of the 14th of August last, I was stopped by a sentinel near Corinth, Miss., and informed that my brother was dying at the hospital of the 18th Mo. Light Artillery. I hastened to the place, and found my brother, Dr. P. J. Wardner, instead of dying himself, trying to prevent the life of another man from gushing out, and anxiously waiting for assistance.

A private, Adolph Bion, of Capt. Sheldon's Battery, was lying upon a cot in the open air: his face pallid, and his lips and tongue bloodless. A flat compress had been placed over the region of the right carotid, and a roller applied *around* the neck, by a surgeon, unknown to me, at the time the patient received his injury. There was still profuse hæmorrhage: his bed was saturated with blood, and he had lost very much before reaching his camp. His pulse was scarcely perceptible. My brother, who had been with him a short time, was partially suppressing the blood, while waiting for aid.

I immediately cut away the bandage, and found the wound largely distended by clots of blood that had been retained by the compress. A part of the clot was removed, and pressure made with the finger below the wound, for the time with entire success. The pulse at times was not perceptible. At the end of an hour he had somewhat revived. I then placed a small-sized hard roller over the artery, and laid one end of a stick of wood upon it, for pressure; and leaving two men to watch him, I left him, after giving direction to be notified if he should be still alive in the morning.

Accordingly, I was informed that no bleeding had occurred, and that my patient was much better; but in half an hour afterwards, a messenger came at full speed, with the news that the man was bleeding "*like a stuck hog.*"

I saw him in all haste; he had made an effort to move his head and had displaced the pressure. Syncope had occurred, and the flow mostly stopped.

The wound was made by the bottom of a brandy bottle in a *row.*

Being prepared for the operation, I at once enlarged the wound, and emptied it of clots, with which it was largely distended. The gush of blood at this moment was truly frightful. The wound was near the division of the common carotid.

Syncope again occurred; and with the able assistance of Assist. Surgeons E. L. Feehon, 1st Mo. Light Artillery, and A. B Brown, 22d Ohio Infantry, I seized upon the moment to make the necessary dissection. The artery was raised out of its sheath with the common ligature needle and tied at the crossing of the *omo-hyoid.* He revived to be conscious, but no hope was entertained of his recovery.

The ligature was not applied above the wound for the following reasons:—

1st. It would have required careful dissection in the carotid space, which the immediate safety of the patient positively forbade.

2d. In case of recovery, it was considered probable that adhesive inflammation and occlusion would take place before the circulation would gain sufficient force to produce hæmorrhage from the opposite side.

3d. That the patient might have time to gain sufficient strength to endure the operation, should it become necessary.

He was conveyed upon a stretcher to a tent near Division Head-Quarters, where I had the opportunity of seeing him daily. The first week after the operation there was a low grade of irritative fever, with a strong tendency to typhoid. But upon the free use of tonics, wine, and nourishing diet, he soon began to mend. The ligature came away on the *eleventh* day.

At the end of five weeks, he was able to stand up, and walk a little with help. On the sixth week, it became necessary to move him to the General Hospital at Corinth, on account of the movement upon Iuca. He was taken in a lumber wagon, and jolted across half a mile of corduroy road in a sitting posture. This produced a good deal of arterial excitement. The night following he had an attack of hæmorrhage from the wound, and a recurrence of the same for four succeeding nights. I learned from the attendants, that arterial excitement, a flushed face, and distended bloodvessels preceded the attack.

On returning from the battle of Iuca, I called Dr. J. G. T. Halston, Medical Director, in consultation. It was determined to trust the case to the use of styptics, low diet, and verat. viride. A tent was introduced deep into the opening, and saturated with *liquor ferri per sulphatis.* This was allowed to remain till i was discharged by suppuration.

This treatment proved successful. The hæmorrhage did not return. The man gradually recovered, and the wound entirely healed.

On the last of October, I saw him walking about the streets of Corinth, "*all right,*" except that he had not recovered his usual strength. He was awaiting his discharge from the service, and longing to be on his way to his native Switzerland.

To me this case is a very interesting one, and the result equally as satisfactory.

Query.—Was it justifiable to omit the ligature above the wound?

Very respectfully, your obedient servant,

H. WARDNER.

